# Docetaxel plus ramucirumab with primary prophylactic pegylated-granulocyte-colony stimulating factor for pretreated non-small cell lung cancer

**DOI:** 10.18632/oncotarget.25578

**Published:** 2018-06-12

**Authors:** Akito Hata, Daijiro Harada, Chiyuki Okuda, Reiko Kaji, Yoshio Masuda, Yoshika Takechi, Toshiyuki Kozuki, Naoyuki Nogami, Nobuyuki Katakami

**Affiliations:** ^1^ Department of Medical Oncology, Kobe City Medical Center General Hospital, Kobe, Japan; ^2^ Department of Pharmacy, Kobe City Medical Center General Hospital, Kobe, Japan; ^3^ Department of Thoracic Oncology, National Hospital Organization Shikoku Cancer Center, Matsuyama, Japan; ^4^ Department of Pharmacy, National Hospital Organization Shikoku Cancer Center, Matsuyama, Japan

**Keywords:** docetaxel, ramucirumab, febrile neutropenia, pegylated-granulocyte-colony stimulating factor

## Abstract

**Purpose:**

The aim of our study was to evaluate the efficacy and safety of docetaxel plus ramucirumab with primary prophylactic pegylated (PEG)-granulocyte-colony stimulating factor (G-CSF) for pretreated non-small cell lung cancer (NSCLC).

**Results:**

Sixty-one pretreated NSCLC patients underwent docetaxel plus ramucirumab. Primary prophylactic PEG-G-CSF was performed in 52 (85%) patients (prophylactic group). No febrile neutropenia (FN) (0%) was confirmed in 52 prophylactic group patients, whereas FN was observed in 3 (33%) of 9 non-prophylactic group patients. Among prophylactic group, median lines of prior therapy was 2 (range, 1–9). Median cycles of docetaxel plus ramucirumab was 3 (range, 1–25) (9 and 3 cases moved to ramucirumab and docetaxel monotherapies, respectively). Response rate and disease control rate were 30.8% and 73.1%, respectively. Median progression-free survival was 4.5 (95% confidence interval [CI], 3.0–6.6) months. Median overall survival was 11.4 (95% CI, 8.0–13.9) months. Six (11.5%) patients had grade 3/4 neutropenia. Observed grade 3 (incidence ≥10%) adverse event (AE) was oral mucositis (13.5%). There were no grade 4/5 non-hematological AEs.

**Conclusions:**

Our study demonstrated the efficacy and safety of docetaxel plus ramucirumab with PEG-G-CSF in clinical practice. Primary prophylactic PEG-G-CSF could markedly reduce incidence of FN.

**Methods:**

We retrospectively reviewed medical records of pretreated NSCLC cases who had received docetaxel plus ramucirumab in our departments.

## INTRODUCTION

Systemic chemotherapy is the standard treatment for metastatic advanced non-small cell lung cancer (NSCLC). Current revolutionally advancement of molecular targeted therapies and immunotherapies has improved prognosis of NSCLC patients. Epidermal growth factor receptor (EGFR)-tyrosine kinase inhibitors (TKIs) or anaplastic lymphoma kinase (ALK)-TKIs exhibited remarkable efficacies over platinum-doublet cytotoxic chemotherapies in patients harboring EGFR gene mutations or ALK gene fusions [1–5]. Furthermore, pembrolizumab, anti-programmed death-1 (PD-1) antibody has demonstrated superior overall survival over platinum-doublets in first-line setting of NSCLC patients with PD-ligand 1 (PD-L1) tumor proportion score ≥50% [6]. Despite an initial dramatic response and durable progression-free survival (PFS) using these agents, progressive disease (PD) is inevitable in most patients. Therefore, second-line chemotherapy is still important to salvage patients after PD on a first-line chemotherapy.

Docetaxel was the standard regimen in second-line chemotherapy for pretreated NSCLC. Although a lot of agents, or those in combination with docetaxel, were compared to docetaxel monotherapy, no agents or combination therapies proved superior to docetaxel monotherapy. Eventually, immunotherapies with PD-1/PD-L1 inhibitors such as nivolumab, pembrolizumab, and atezolizumab have shown superior survival benefit over docetaxel monotherapy [7–10]. These immunotherapies give a strong survival benefit to responders, but disease control rate is relatively low, and early death is a serious concern in non-responders [11].

Ramucirumab is a fully human immunoglobulin G1 monoclonal antibody that binds with high affinity to vascular endothelial growth factor (VEGF) receptor-2, preventing VEGF binding and activation [12]. A randomized, phase III REVEL trial demonstrated that additional ramucirumab to docetaxel prolonged OS in NSCLC patients who had progressed after first-line platinum-based therapy [13]. In this trial, superior response rate (RR) and PFS translated to OS benefit with a high disease control rate (DCR). A similarly designed Japanese randomized phase II trial comparing docetaxel plus ramucirumab with docetaxel monotherapy showed that effectiveness of additional ramucirumab was comparable to those of REVEL study [14]. On the other hand, a safety concern was suggested. Febrile neutropenia (FN) was confirmed in 34% of these Japanese patients in docetaxel plus ramucirumab arm. This high incidence of FN presents an important clinical problem for docetaxel plus ramucirumab, used routinely in Japanese clinical practice.

The American Society of Clinical Oncology practice guideline recommends primary prophylactic granulocyte-colony stimulating factor (G-CSF) when the risk of FN is approximately 20% or higher [15]. We thus consider primary prophylactic G-CSF to be suitable in docetaxel plus ramucirumab therapy for Japanese patients. Pegylated (PEG)-G-CSF demonstrated reduction of FN incidence administered once a cycle in many types of cancers. PEG-filgrastim is a polyethylene glycol-derived form of filgrastim. Based on the features of PEG-conjugated proteins, PEG-filgrastim was designed to prolong the half-life of filgrastim and to decrease the number of injections. A Japanese double-blind, placebo-controlled, randomized phase III trial of PEG-filgrastim in 343 breast cancer patients receiving docetaxel and cyclophosphamide chemotherapy showed that the incidence of FN was significantly lower in the PEG-filgrastim group than in the placebo group (1.2% vs. 68.8 %, *P <* 0.001) [16]. In NSCLC patients, incidence of FN could also be reduced by PEG-G-CSF.

Based on the above background, we actively use primary prophylactic PEG-G-CSF in our clinical practice on docetaxel plus ramucirumab therapy. The aim of this retrospective study was to evaluate the efficacy and safety of docetaxel plus ramucirumab with primary prophylactic PEG-G-CSF support for pretreated NSCLC in Japanese clinical practice.

## RESULTS

### Patient characteristics

**Figure 1:**
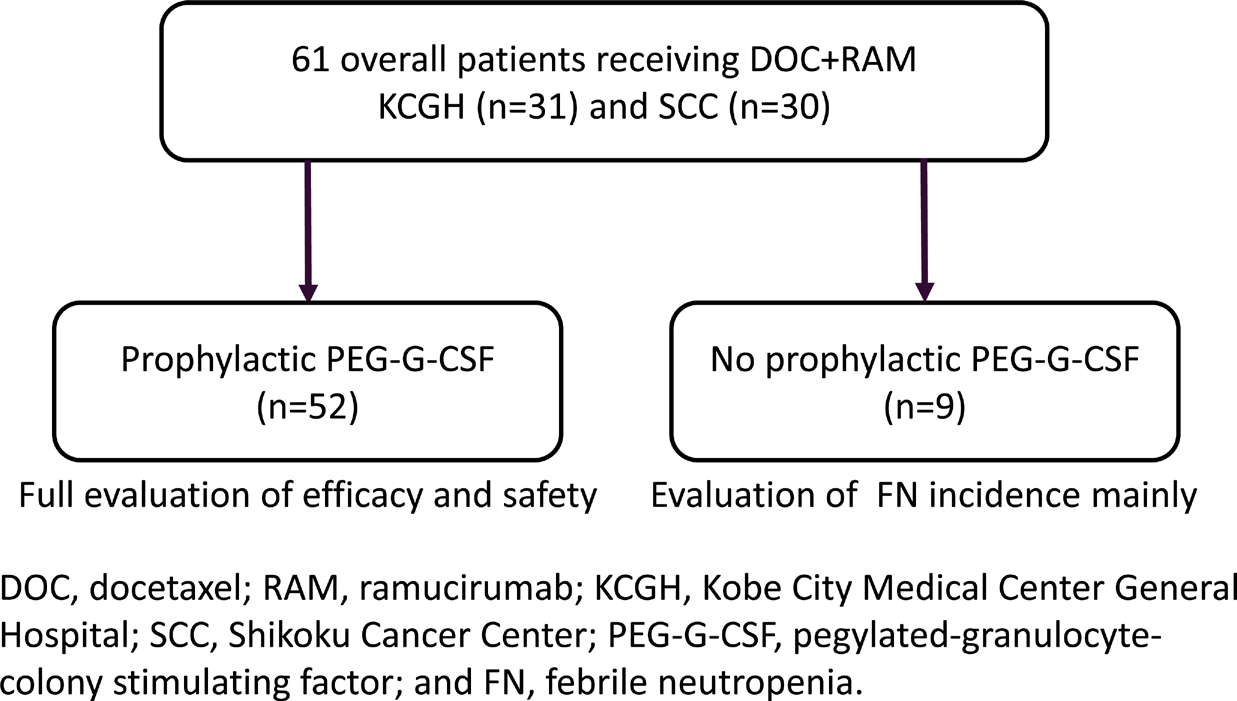
Study flow chart

Study flow chart is shown in Figure 1. Between June 2013 and April 2018, 61 pretreated NSCLC patients received docetaxel plus ramucirumab at our departments (31 Department of Medical Oncology, Kobe City Medical Center General Hospital [KCGH] and 30 Department of Thoracic Oncology, Shikoku Cancer Center [SCC]). We fully evaluated the efficacy and safety of docetaxel plus ramucirumab in 52 (85%) patients receiving primary prophylactic PEG-G-CSF (prophylactic group). In remaining 9 (15%) patients without primary prophylactic PEG-G-CSF (non-prophylactic group), incidence of FN was mainly examined. Patient characteristics are shown in Table 1. Among prophylactic group, median age was 64 (range, 47–79). Male (65%), smoker (73%), and good Eastern Cooperative Oncology Group performance status (PS) (0/1) (87%) were dominant. Fifteen (29%) were *EGFR* mutant (no *ALK*-fusioned). Median number of prior regimens was 2 (range, 1–8). Prior bevacizumab was administered in 21 (40%) patients. Prior EGFR-TKIs were prescribed in all 15 *EGFR*-mutant patients, and two wild-type patients underwent erlotinib. All patients received docetaxel plus ramucirumab after failure of platinum-doublets. Prior taxanes were administered in 28 (54%) patients. Patient characteristics of non-prophylactic group were similar to prophylactic group.

**Table 1:** Patient characteristics

Characteristics	Prophylactic group (*n* = 52)	Non-prophylactic group (*n* = 9)
Median age, years (range)	64 (47–79)	67 (61–76)
Gender		
Male	34 (65%)	5 (56%)
Female	18 (35%)	4 (44%)
Smoking history		
Never	14 (27%)	4 (44%)
Ever	38 (73%)	5 (56%)
Performance status		
0/1	3/42 (87%)	1/8 (100%)
2/3	5/2 (13%)	0/0 (0%)
Histology		
Adenocarcinoma	35 (67%)	7 (78%)
Squamous/Large	16/1 (33%)	2/0 (22%)
*EGFR* mutation		
Mutant	15 (29%)	3 (33%)
Wild	37 (71%)	6 (67%)
Prior regimens, median (range)	2 (1–8)	1 (1–9)
Prior bevacizumab		
Administered	21 (40%)	3 (33%)
None	31 (60%)	6 (67%)
Prior EGFR-TKIs		
Administered	17 (33%)	3 (33%)
None	35 (67%)	6 (67%)
Prior platinum doublets		
Administered	52 (100%)	9 (100%)
None	0 (0%)	0 (0%)
Prior taxanes		
Administered	28 (54%)	5 (56%)
None	24 (46%)	4 (44%)

Abbreviations: EGFR, epidermal growth factor receptor; TKI, tyrosine kinase inhibitor.

### Incidence of febrile neutropenia

No FN (0%: 95% confidence interval [CI], 0–6.9%) was confirmed in 52 prophylactic group patients, whereas FN was observed in 3 (33%: 95% CI, 7.5–70.1%) of 9 non-prophylactic group patients.

### Efficacy in prophylactic group

**Figure 2:**
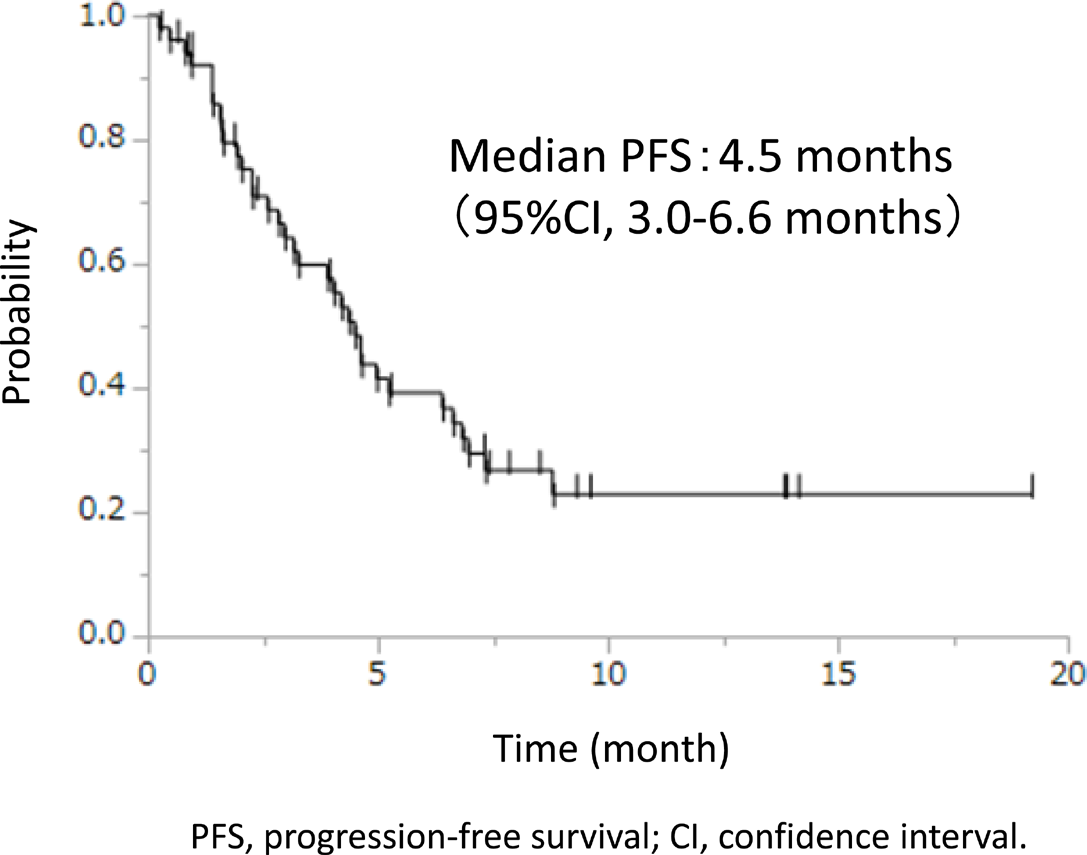
Progression-free survival

One (1.9%) complete response (CR), 15 (28.8%) partial response (PR), 22 (42.3%) stable disease (SD), 12 (23.1%) PD, and 2 (3.8%) not evaluable were confirmed, resulting in RR of 30.8% (95% CI, 18.7–45.1%) and DCR of 73.1% (95% CI, 60.4–86.4%), respectively. The median PFS was 4.5 (95% CI, 3.0–6.6) months (Figure 2), and the median OS was 11.4 (95% CI, 8.0–13.9) months (referential results because docetaxel plus ramucirumab was administered at multiple lines).

### Safety in prophylactic group

Table 2 summarizes adverse events (AEs) in 52 patients with prophylactic PEG-G-CSF. Hematological AEs ≥grade 3: 6 (11.5%) neutropenia; 3 (5.8%) anemia; and 2 (3.8%) thrombocytopenia were recorded. Non-hematological AEs ≥grade 3: 7 (13.5%) oral mucositis; 4 (7.7%) anorexia; 2 (3.8%) interstitial lung disease (ILD); 1 allergy (1.9%); 1 (1.9%) hand-foot syndrome; and 1 (1.9%) neurotoxicity were observed. Ramucirumab-associated AEs ≥grade 3: 1 (1.9%) brain tumor hemorrhage; 1 (1.9%) gastrointestinal bleeding; and 1 (1.9%) venous thrombosis were found. Neither grade 4 nor 5 non-hematological AEs were confirmed.

**Table 2:** Adverse events in patients with prophylactic PEG-G-CSF (*n* = 52)

Adverse event	Grade 1/2 (*n*)	Grade 3/4 (*n*)	≥ grade 3 (%)	All (%)
Hematological				
Neutropenia	10	6	11.5	30.8
Anemia	26	3	5.8	55.8
Thrombocytopenia	12	2	3.8	26.9
Febrile neutropenia	-	0	0	0
Non-hematological				
Anorexia	23	4	7.7	51.9
Malaise	18	0	0	34.6
Oral mucositis	19	7	13.5	50.0
Interstitial pneumonia	0	2	3.8	3.8
Neurotoxicity	4	1	1.9	9.6
Allergy	0	1	1.9	1.9
Hand-foot syndrome	0	1	1.9	1.9
Ramucirumab-associated				
Hemoptysis	3	0	0	5.8
Epistaxis	7	0	0	13.5
Hypertension	7	0	0	13.5
Proteinuria	14	0	0	26.9
Venous thrombosis	0	1	1.9	1.9
Brain hemorrhage	0	1	1.9	1.9
Gastrointestinal bleeding	0	1	1.9	1.9

No grade 4/5 adverse events

Abbreviations: PEG-G-CSF, pegylated-granulocyte-colony stimulating factor.

### Administration of docetaxel and ramucirumab in prophylactic group

Docetaxel was administered at 60 mg/m^2^ in 44 (85%) patients and at 50 mg/m^2^ in 8 (15%) patients (dose reduction was performed at physicians’ discretion). Median cycles of docetaxel and ramucirumab was 3 (range, 1–25). Fourteen (29%) patients received more than 6 cycles of the therapy. Three (6%) and 9 (17%) patients moved to docetaxel and ramucirumab monotherapy, respectively, because of intolerable toxicities or physicians’ discretion.

### Characteristics of febrile neutropenia patients

Table 3 shows data on three patients who suffered from FN. These three patients did not receive prophylactic PEG-G-CSF. All patients had good PS (ECOG PS: 1), but experienced grade 4 neutropenia. Two of 3 patients were aged ≥75. Prior bevacizumab was administered in one patient. Two patients were collected from SCC and one was from KCGH.

**Table 3:** Characteristics of febrile neutropenia patients (*n* = 3)

Age	Sex	His	PS	*EGFR*	Smoking	DOC dose	PEG- G-CSF	Line of DOC + RAM	Prior Bev	Institute	Response	PFS	Adverse events (grade ≥2)
63	M	Ad	1	Wild	Former	60	−	2nd	+	SCC	SD	1.9	Neutropenia (G4)
75	M	Ad	1	Wild	Former	60	−	2nd	−	SCC	PD	1.8	Neutropenia (G4), anemia (G2), thrombocytopenia (G2), anorexia (G2), mucositis (G2)
76	M	Ad	1	L858R	Never	50	−	4th	−	KCGH	NE	1.0	Neutropenia (G4), mucositis (G2)

Abbreviations: His, histology; PS, performance status; EGFR, epidermal growth factor receptor; DOC, docetaxel; PEG-G-CSF, pegylated-granulocyte-colony stimulating factor; RAM, ramucirumab; Bev, bevacizumab; SCC, Shikoku Cancer Center; KCGH, Kobe City Medical Center General Hospital; PFS, progression-free survival; M, male; F, female; Ad, adenocarcinoma; SD, stable disease; PD, progressive disease; NE, not evaluable; G, grade; ILD, interstitial lung disease.

### Characteristics of elderly patients aged ≥75

Table 4 shows data on elderly patients aged ≥75 (*n* = 11). Initial dose of docetaxel was reduced to 50 mg/m^2^ in four cases at physicians’ discretion. No FN was confirmed in all nine cases receiving PEG-G-CSF support, whereas two cases without PEG-G-CSF had FN. Four of these 11 patients achieved PR. AEs ≥grade 2; 6 mucocitis; 5 anemia; 3 thrombocytopenia; 2 neutropenia; 1 anorexia; 1 malaise; 1 hand-foot syndrome; 1 ILD; and 1 hypertension were observed.

**Table 4:** Characteristics of elderly patients aged ≥75 (*n* = 11)

Age	Sex	His	PS	*EGFR*	Smoking	DOC dose	PEG- G-CSF	Line of DOC + RAM	FN	Response	PFS	Adverse events (grade ≥2)
75	M	Ad	1	Wild	Former	60	+	2nd	−	PR	2.4	Anemia (G2)
75	F	Sq	1	Wild	Never	50	+	7th	−	SD	4.4	Thrombocytopenia (G3)
75	M	Ad	1	Wild	Former	60	−	2nd	+	PD	1.8	Neutropenia (G4), anemia (G2), thrombocytopenia (G2), anorexia (G2), mucositis (G2)
76	M	La	1	Wild	Former	60	+	3rd	−	SD	2.6	Anemia (G2), thrombocytopenia (G2), anorexia (G2), mucositis (G2), malaise (G2), hand-foot syndrome (G3)
76	M	Ad	1	L858R	Former	60	+	7th	−	SD	2.1	Anemia (G2)
76	M	Ad	1	L858R	Never	50	-	4th	+	NE	1.0	Neutropenia (G4), mucositis (G2)
77	M	Sq	1	Wild	Former	60	+	3rd	−	PR	19.2+	Hypertension (G2)
78	M	Sq	1	Wild	Former	60	+	3rd	−	PR	7.3+	Mucositis (G2)
78	F	Ad	1	Del-19	Never	50	+	3rd	−	PR	6.6	Anemia (G2), ILD (G3)
79	F	Ad	1	Wild	Never	50	+	3rd	−	SD	7.4+	Mucositis (G2)
79	F	Ad	2	Wild	Never	60	+	2nd	−	SD	8.5 +	Mucositis (G2)

Abbreviations: His, histology; PS, performance status; EGFR, epidermal growth factor receptor; DOC, docetaxel; PEG-G-CSF, pegylated-granulocyte-colony stimulating factor; RAM, ramucirumab; FN, febrile neutropenia; PFS, progression-free survival; M, male; F, female; Ad, adenocarcinoma; Sq, squamous cell carcinoma; La, large cell carcinoma; PR, partial response; SD, stable disease; PD, progressive disease; NE, not evaluable; G, grade; ILD, interstitial lung disease.

## DISCUSSION

To the best of our knowledge, this is the first report to investigate the efficacy and safety of docetaxel plus ramucirumab with primary prophylactic PEG-G-CSF support in pretreated NSCLC. Our study demonstrated 0% of FN incidence by primary prophylactic PEG-G-CSF support in docetaxel plus ramucirumab therapy for Japanese patients with pretreated NSCLC. FN incidence of REVEL and Japanese phase II study in docetaxel plus ramucirumab were 13.3% and 34.2%, respectively [13, 14]. Our study suggests high potency of primary prophylactic PEG-G-CSF support to prevent FN in docetaxel plus ramucirumab therapy.

The RR and DCR in prophylactic group were 30.8% and 73.1%, respectively. These results were comparable to REVEL (23% and 64%) and Japanese phase II study (28.5% and 78.9%) in each docetaxel plus ramucirumab arm [13, 14]. The median PFS was 4.5 months, which was also similar to that of REVEL (4.5 months) and Japanese phase II study (5.2 months) [13, 14]. Although our study population included relatively many heavily pretreated patients, primary prophylactic PEG-G-CSF support improved the safety while maintaining the effectiveness in docetaxel plus ramucirumab therapy.

Neither grade 4 nor 5 non-hematological AEs were confirmed, owing to primary prophylactic PEG-G-CSF support, with neutropenia ≥grade 3 only observed in 6 (11.5%) patients. Some ramucirumab-associated AEs ≥grade 3 were confirmed: 1 (1.9%) brain tumor hemorrhage; 1 (1.9%) gastrointestinal bleeding; and 1 (1.9%) venous thrombosis. Incidences of these VEGF-associated AEs were not high, compared to historical data of docetaxel plus ramucirumab therapy [13, 14]. Oral mucositis was the only non-hematological AEs ≥grade 3 observed more than 10% of patients. Physicians should beware of this AE for heavily pretreated patients receiving docetaxel plus ramucirumab therapy in clinical practice.

The study populations included 11 patients aged ≥75. No FN was confirmed in all nine cases receiving PEG-G-CSF support, whereas two cases without PEG-G-CSF had FN. Moreover, four of these nine patients achieved PR, and general AEs were tolerable. Although docetaxel dose was reduced to 50 mg/m^2^ in a 76 year-old male, FN was confirmed with grade 4 neutropenia. Administration of PEG-G-CSF seems a better option than dose reduction. PEG-G-CSF support might be much more reasonable for elderly patients receiving docetaxel plus ramucirumab. Indeed, a multicenter phase II trial to evaluate the efficacy and safety of docetaxel plus ramucirumab with PEG-G-CSF support for chemo-naïve elderly NSCLC patients is ongoing (West Japan Oncology Group 9416L).

Our study was retrospective and small sample size, including several limitations. Regular measurement of temperature was not routinely carried out. FN incidence might have been estimated lower. However, FN is generally considered as an oncology emergency, and clinically relevant FN must have been detected. Tumor response and PFS were evaluated using the Response Evaluation Criteria in Solid Tumors (RECIST), but durations of CT scans depended on doctors in charge. These variable durations could be a bias of our study. However, purpose of chemotherapy for pretreated patients was not response, but living longer with better quality of life (QOL). Subset analysis of the Japanese phase II trial suggested a possible association between FN occurrence and QOL deterioration [14, 17]. PEG-G-CSF support could maintain QOL in pretreated patients receiving docetaxel plus ramucirumab therapy.

In conclusion, our study demonstrated 0% of FN incidence by primary prophylactic PEG-G-CSF support in docetaxel plus ramucirumab therapy for Japanese patients with pretreated NSCLC. Primary prophylactic PEG-G-CSF could markedly reduce incidence of FN. Our study populations included many heavily pretreated patients, while docetaxel plus ramucirumab showed notable efficacy and safety. Further studies are warranted to confirm the effectiveness of primary prophylactic PEG-G-CSF support in docetaxel plus ramucirumab therapy.

## METHODS

### Patients

This is a retrospective study of two institutes. We screened all NSCLC patients to identify cases who had received docetaxel plus ramucirumab in Department of Medical Oncology, KCGH and Department of Thoracic Oncology, SCC. Patients’ results were collected using electric medical and radiographic records to take clinical information: age; gender; smoking history; PS; histology; *EGFR*-mutation or *ALK*-fusion status; prior therapies before docetaxel plus ramucirumab; and clinical course details. We evaluated the RR, DCR, PFS, OS, and safety, especially FN. This study was approved by the institutional review board of each institute.

### Treatment

Intravenous docetaxel (60 mg/m^2^, day 1) plus intravenous ramucirumab (10 mg/kg, day 1) with subcutaneous PEG-G-CSF (3.6 mg, day 2) every 3 weeks was administered until progression or unacceptable toxicities. Initial docetaxel dose of some cases was reduced to 50 mg/m^2^ by the discretion of physicians in charge. In cases with intolerable toxicities, docetaxel dose was reduced to 50 mg/m^2^, 40 mg/m^2^, or 30 mg/m^2^. Furthermore, docetaxel or ramucirumab monotherapy was administered when intolerable toxicities occurred but clinical benefit was obtained by each drug.

Tumor evaluations were performed every 6–9 weeks with computed tomography.

### Statistical analysis

Tumor response was evaluated in accordance with the RECIST (version 1.1). The DCR was defined as the rate of CR/PR + SD ≥6 weeks in our study. The PFS was calculated from the date of therapy initiation to disease progression or death. The OS was calculated from the date of therapy initiation to death, and censored at the date of last visit for patients whose deaths could not be confirmed. PFS and OS were analyzed using the Kaplan-Meier method to estimate the median points with 95% CI. Toxicity was assessed according to the National Cancer Institute Common Terminology Criteria for Adverse Events (version 4.0). The statistical analyses were performed using JMP 12 (SAS Institute, Inc., Cary, NC, USA).
